# Diet-derived circulating antioxidants and risk of inflammatory bowel disease: a Mendelian randomization study and meta-analysis

**DOI:** 10.3389/fimmu.2024.1334395

**Published:** 2024-02-21

**Authors:** Menglong Zou, Qiaoli Liang, Wei Zhang, Junyao Liang, Ying Zhu, Yin Xu

**Affiliations:** ^1^ Department of Gastroenterology, The First Hospital of Hunan University of Chinese Medicine, Changsha, Hunan, China; ^2^ Department of Oncology, Doumen Qiaoli Hospital of Traditional Chinese Medicine, Zhuhai, China

**Keywords:** diet-derived circulating antioxidants, inflammatory bowel disease, causal relationship, Mendelian randomization, meta-analysis

## Abstract

**Background:**

Previous studies have shown conflicting results regarding the impact of circulating antioxidants on the risk of inflammatory bowel disease (IBD). In this study, our intent was to investigate the causal relationship between circulating antioxidants and IBD using Mendelian randomization (MR).

**Methods:**

Instrumental variables for absolute circulating antioxidants (ascorbate, retinol, lycopene, and β-carotene) and circulating antioxidant metabolites (α-tocopherol, γ-tocopherol, ascorbate, and retinol) were screened from published studies. We obtained outcome data from two genome-wide association study (GWAS) databases, including the international inflammatory bowel disease genetics consortium (IIBDGC, 14,927 controls and 5,956 cases for Crohn’s disease (CD), 20,464 controls and 6,968 cases for ulcerative colitis (UC), and 21,770 controls and 12,882 cases for IBD) and the FinnGen study (375,445 controls and 1,665 cases for CD, 371,530 controls and 5,034 cases for UC, and 369,652 controls and 7,625 cases for IBD). MR analysis was performed in each of the two databases and those results were pooled using meta-analysis to assess the overall effect of exposure on each phenotype. In order to confirm the strength of the findings, we additionally conducted a replication analysis using the UK Biobank.

**Results:**

In the meta-analysis of the IIBDGC and FinnGen, we found that each unit increase in absolute circulating level of retinol was associated with a 72% reduction in the risk of UC (OR: 0.28, 95% CI: 0.10 to 0.78, *P*=0.015). The UC GWAS data from the UK Biobank also confirmed this causal relationship (OR: 0.99, 95% CI: 0.97 to 1.00, *P*=0.016). In addition, there was suggestive evidence that absolute retinol level was negatively associated with IBD (OR: 0.41, 95% CI: 0.18 to 0.92, *P*=0.031). No other causal relationship was found.

**Conclusion:**

Our results provide strong evidence that the absolute circulating level of retinol is associated with a reduction in the risk of UC. Further MR studies with more instrumental variables on circulating antioxidants, especially absolute circulating antioxidants, are needed to confirm our results.

## 1 Introduction

Inflammatory bowel disease (IBD) is an immune-mediated disease (1). The major clinical symptoms of IBD include abdominal pain, hematochezia, and diarrhea (1). Two major subtypes of IBD are ulcerative colitis (UC) and Crohn’s disease (CD) (2). UC is characterized by an inflammation limited to the colon and the rectum, beginning in the rectum and extending to the proximal colon (3, 4). In contrast, CD can impact any part of the digestive system, with a particular focus on the terminal ileum. Evidence from previous studies suggests that oxidative stress plays a critical in the development of IBD (5–7). Previous Mendelian randomization (MR) analysis also identified oxidative stress-related genetic risk loci for CD (8). New insights into the treatment of IBD may be gained by elucidating the causal role of antioxidants in the disease.

Oxidative stress represents an imbalance between antioxidants and pro-oxidants. The infiltration of activated immune cells into intestinal mucosal tissue generates reactive oxygen species (ROS), resulting in a shift towards pro-oxidants and away from antioxidants (9). The excessive accumulation of ROS disrupts cellular homeostasis. To maintain cellular homeostasis, antioxidants must balance pro-oxidant activity, which is achieved through four lines of defense (10). Superoxide dismutase (SOD) is an antioxidant enzyme that constitutes the first line of defense against ROS. Its primary function is to prevent the formation of free radicals and neutralize those that have already formed. The second line of defense is free radical scavengers, including ascorbate, carotene, retinol and others, which neutralize free radicals by donating electrons. The third and fourth lines of defense focus on eliminating the damage caused by pro-oxidants at the molecular and cellular levels. Diet-derived antioxidants are known to be the most readily available. Several studies have suggested that ascorbate levels are associated with disease activity in IBD (11, 12). Ascorbate has also been shown to regulate tight junction complexes to restore epithelial barrier integrity (13). However, another retrospective study of 301 participants with IBD found no difference in clinical symptoms between those with and without ascorbate deficiency (14). Hence, the relationship between circulating antioxidants and risk of IBD remains controversial.

MR is a robust method for assessing aetiological inference in epidemiological analysis (15). It introduces the concept of instrumental variables to examine the relationship between genetic variants associated with exposure and disease or trait. This analytical approach uses Mendel’s first and second laws of inheritance, where effect alleles are randomly distributed to the offspring during meiosis. Therefore, MR analysis can significantly reduce the effects of reverse causality and confounding, which are limitations of observational and retrospective studies. Here, we investigated the causal relationship between circulating antioxidants and risk of IBD using MR analysis.

## 2 Materials and methods

### 2.1 Study design

**Figure 1:**
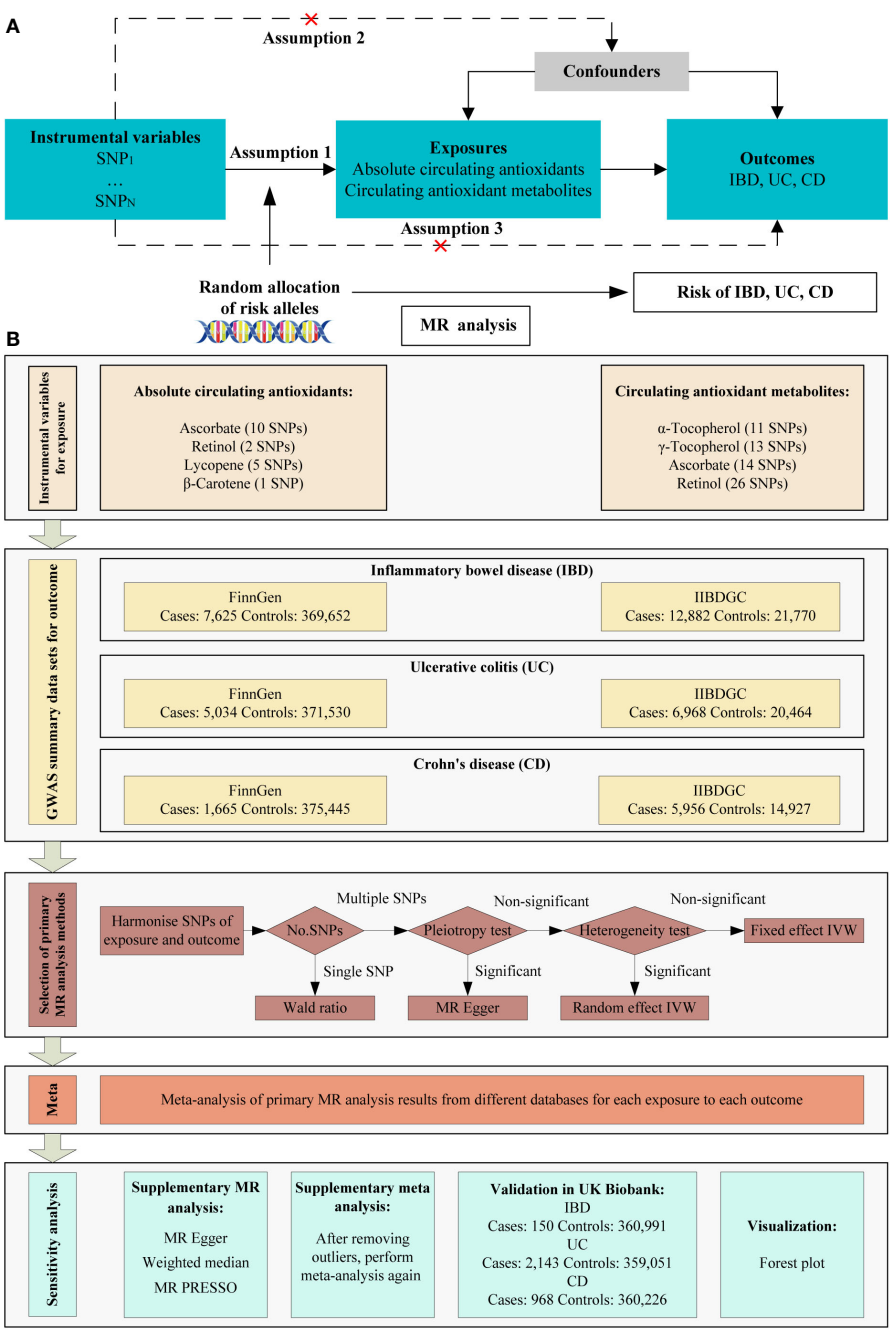
Schematic overview of the MR study design. **(A)** The three main assumptions of MR analysis. **(B)** Schematic overview and framework of the present MR study design. SNP, single-nucleotide polymorphism; MR, Mendelian randomization; IBD: inflammatory bowel disease; UC: ulcerative colitis; CD: Crohn’s disease; IIBDGC: international inflammatory bowel disease genetics consortium; IVW, inverse variance weighted; MR PRESSO, MR Pleiotropy RESidual Sum and Outlier.

Based on genome-wide association study (GWAS) summary data, we used MR analysis to investigate the causal relationship between diet-derived circulating antioxidants (ascorbate, lycopene, retinol, β-carotene, γ-tocopherol, and α-tocopherol) and risk of IBD. For circulating antioxidants, we chose two phenotypes, including absolute circulating antioxidants and circulating antioxidant metabolites. Authentic absolute levels in the blood were defined as absolute circulating antioxidants, while relative concentrations in plasma or serum were defined as circulating antioxidant metabolites (16). Instrumental variables must satisfy the three main assumptions of MR analysis (**Figure 1A**). MR analysis was conducted in each of two European databases, including the FinnGen study (17) and international inflammatory bowel disease genetics consortium (IIBDGC) (18), and the results were pooled using meta-analysis to assess the overall effect of exposure on each phenotype. The framework of the study design is shown in **Figure 1B**. Ethical approval was unnecessary as the study utilized publicly accessible data.

### 2.2 Genetic instrumental variables selection

For absolute circulating antioxidants, single nucleotide polymorphisms (SNPs) were identified from recent large-scale GWAS as instrumental variables for ascorbate, retinol, and β-carotene (*P* < 5 × 10^−8^, r^2^ < 0.001, and kb =10,000). Eleven ascorbate-related SNPs were extracted from recently published GWAS including up to 52,018 individuals (19). After linkage disequilibrium (LD) clumping, one of the eleven SNPs was discarded. Two independent SNPs for retinol were extracted from a GWAS meta-analysis that included two cohort studies with a total of 5,006 individuals (20). GWAS of 2,344 individuals from the Nurses’ Health Study identified two SNPs significantly related to β-carotene (21). One of the two SNPs was discarded after LD clumping. For lycopene, five independent SNPs were identified from a GWAS of 441 older Amish adults (*P* < 5 × 10^−6^, r^2^ < 0.001, and kb =10,000) (22).

For circulating antioxidant metabolites, we used looser thresholds (*P* < 1 × 10^−5^, r^2^ < 0.001, and kb =10,000). In summary, a GWAS of 7,824 adults identified fourteen independent SNPs for ascorbate, thirteen for γ-tocopherol, and eleven for α-tocopherol (23). Twenty-six independent SNPs were identified as instrumental variables for retinol in 1,960 participants (24).

Phenotype scanning was performed using the PhenoScanner database to analyze the association of SNPs with confounders (25). If the association did not reach genome-wide significance, it was retained. The variance (R^2^) in the MR analysis is defined as the proportion of total variance explained by the genetic instrumental variables. The strength of the instrumental variables for circulating antioxidants was assessed by the *F*-statistic.

Cohort information for the GWAS used to extract instrumental variables is presented in **Supplementary Table 1**. Summary information on the instrumental variables is presented in **Table 1**. The variance explained by the instrumental variables ranged from 1.7% to 30.1% for circulating absolute antioxidants and from 6.8% to 21.7% for circulating antioxidant metabolites, with all *F*-statistics greater than 10. Details of the SNPs are listed in **Supplementary Tables 2**, **3**.

**Table 1:** The summary of instrumental variables for diet-derived absolute circulating antioxidants and antioxidant metabolites.

Trait	Sample Size	*P*	LD	No. of SNPs	Explained Variance (R^2^, %)	Unit	PMID
Absolute circulating antioxidants							
Ascorbate	52,018	5 × 10^−8^	0.001	10	1.7	µmol/L	33203707
Lycopene	441	5 × 10^−6^	0.001	5	30.1	µg/dL	26861389
Retinol	5,006	5 × 10^−8^	0.001	2	2.3	µg/L in ln-transformed scale	21878437
β-Carotene	2,344	5 × 10^−8^	0.001	1	4.8	µg/L in ln-transformed scale	23134893
Circulating antioxidant metabolites							
α-Tocopherol	7725	1 × 10^−5^	0.001	11	6.8	log10-transformed metabolites concentration	24816252
γ-Tocopherol	6226	1 × 10^−5^	0.001	13	9.8	log10-transformed metabolites concentration	24816252
Ascorbate	2085	1 × 10^−5^	0.001	14	21.7	log10-transformed metabolites concentration	24816252
Retinol	1960	1 × 10^−5^	0.001	26	20.6	log10-transformed metabolites concentration	28263315

LD: linkage disequilibrium. The R^2^ of each circulating antioxidant was extracted from the original study or calculated based on the following formula: R^2^ = (2×EAF×(1-EAF) ×Beta^2^)/[(2×EAF×(1-EAF) ×Beta^2^) +(2×EAF×(1-EAF) ×N×SE^2^)]. EAF is the effect allele frequency, Beta indicates the estimated genetic effect of SNP, N is sample size, and SE is standard error of the estimated effect

### 2.3 Outcome data sources

Three large databases, including IIBDGC, FinnGen study, and UK Biobank, were used to obtain GWAS data for outcome. The primary analysis used data from the IIBDGC and FinnGen, while the replication analysis used data from the UK Biobank. To make the results more reliable, we prioritized GWAS summary data with strict disease definitions rather than self-reports. The FinnGen study included 369,652 controls and 7,625 cases for IBD, 371,530 controls and 5,034 cases for UC, and 375,445 controls and 1,665 cases for CD, while the IIBDGC included 21,770 controls and 12,882 cases for IBD, 20,464 controls and 6,968 cases for UC, and 14,927 controls and 5,956 cases for CD. There were no strictly defined IBD summary data in the UK Biobank. Therefore, self-reported IBD summary data were used for the replication analysis. Specifically, there were 150 self-reported IBD cases and 360,991 controls, 2,143 main diagnosed UC cases and 359,051 controls, and 968 main diagnosed CD cases and 360,226 controls. Details of outcome data are shown in **Supplementary Table 4**. We removed the SNPs that were not present in the outcome GWAS summary data. Incompatible or palindromic SNPs also were discarded.

### 2.4 Statistical analysis

The primary MR analysis method is shown in **Figure 1B**. Firstly, we harmonized the exposure and outcome information using effect allele of SNPs. In cases where only one SNP was retained, we used the Wald ratio as the primary analysis method. When multiple SNPs were available, pleiotropy was assessed using the MR Egger intercept test. When pleiotropy was present, MR Egger served as the primary method for MR analysis; otherwise, inverse variance weighting (IVW) was utilized. Heterogeneity was then assessed using the *P*-value of the Cochran’s Q statistic. The random effect IVW model was used when heterogeneity was present, otherwise the fixed effect IVW model was used. For tests of pleiotropy and heterogeneity, *P <*0.05 was defined as significant.

The MR analysis was conducted in each of two European databases, including the FinnGen and the IIBDGC, and the results were pooled using meta-analysis to assess the overall effect of each specific outcome. The *I^2^* statistic was calculated using Cochran’s Q-test to quantify the heterogeneity between estimates. The random effect meta-analysis model was used when heterogeneity was present, otherwise the fixed effect model was used.

To evaluate the strength of the findings, a range of sensitivity analyses were performed. Firstly, supplementary MR analysis with different assumptions was used to validate the causality inferred by the primary MR analysis. The MR Egger method calculates pleiotropy-robust causal effect values from standard errors (number of SNPs > 2). If at least half of the weights are counted using valid instrumental variables (number of SNPs > 2), then the weighted median can present a valid causal estimate. The MR Pleiotropy Residual Sum and Outlier (MR PRESSO) method was utilized for identifying and rectifying any outliers. Finally, significant and suggestive evidence was validated using GWAS summary data from UK Biobank.

There were three outcomes in our primary MR analysis. The Bonferroni correction threshold of *P* < 0.017 (0.05/3 outcomes) was considered statistically significant to account for the fact that multiple testing may lead to false-positive results. Suggestive evidence was characterized as a *P*-value ranging from 0.05 to 0.017. Replication MR analysis was performed based on significant and suggestive evidence from the primary analysis, with *P* < 0.05 defined as significant. The study utilized the R software (version 4.2.2) to conduct MR analysis with the assistance of the TwoSampleMR package (version 0.5.6). Meta-analysis was performed using the meta package (version 6.5.0).

## 3 Results

### 3.1 Absolute circulating antioxidants and inflammatory bowel disease

**Figure 2:**
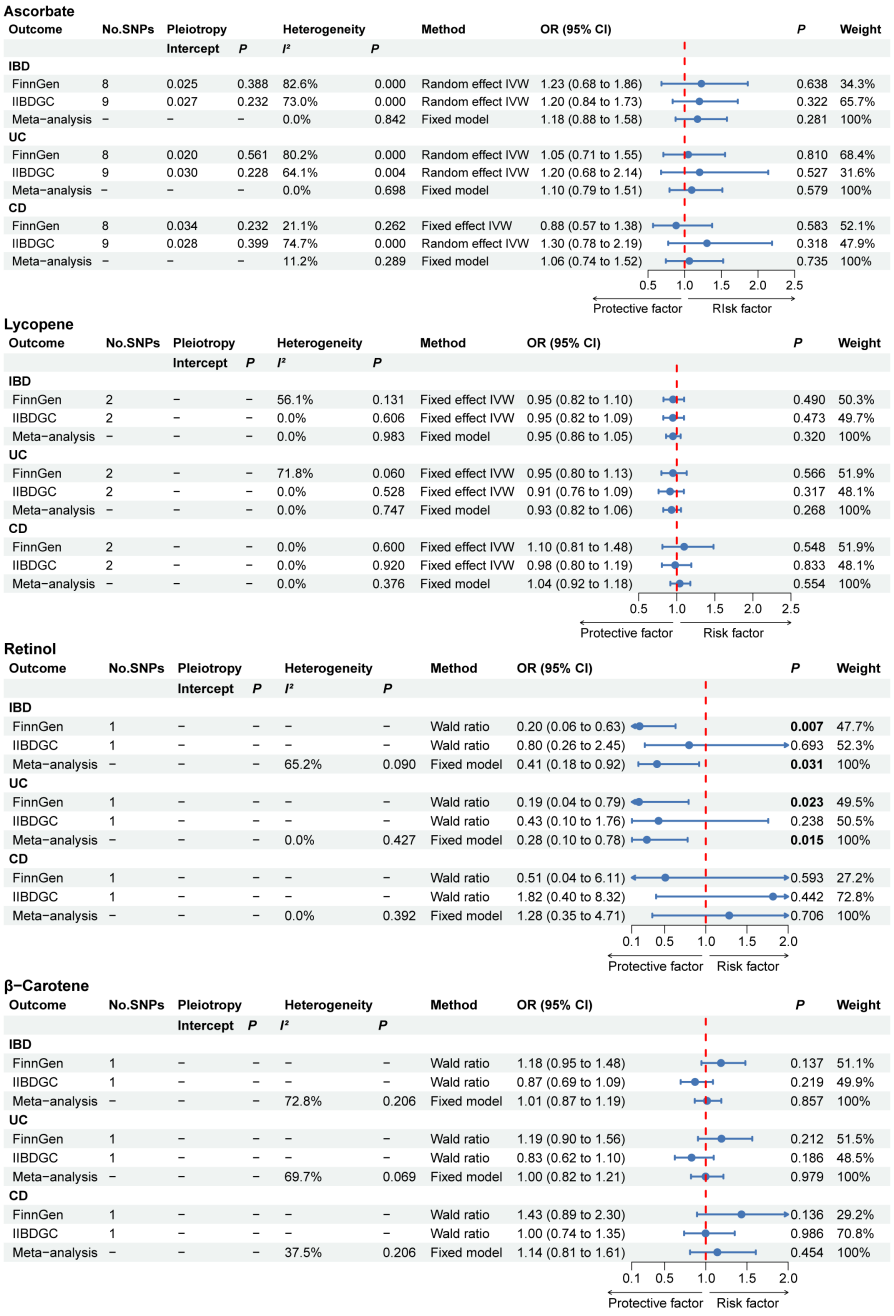
The primary MR analyses results of the causal effects of absolute circulating antioxidant levels on IBD, UC, and CD. Significant and suggestive results highlighted in bold. “-” represents not applicable. IBD, inflammatory bowel disease; UC, ulcerative colitis; CD, Crohn’s disease.

The main findings of absolute circulating antioxidants in the MR results are shown in the forest plot in **Figure 2**. The MR Egger intercept test showed a *P* value ranging from 0.228 to 0.561 for ascorbate, suggesting the absence of horizontal pleiotropy. No association between the absolute circulating level of ascorbate and the risk of IBD (including UC and CD) in either the FinnGen or IIBDGC. For IBD, UC and CD, the overall effect per 1 µmol/L ascorbate was 1.18 (95% CI 0.88 to 1.58), 1.10 (95% CI 0.79 to 1.51) and 1.06 (95% CI 0.71 to 1.52), respectively. Absolute circulating level of lycopene was also not associated with risk of IBD, UC or CD in any database, with pooled OR per 1 µg/dL lycopene ranging from 0.93 (95% CI: 0.82 to 1.06) for UC to 1.04 (95% CI: 0.92 to1.18) for CD. The risk of IBD and UC was negatively associated with the absolute circulating concentration of retinol in the FinnGen study. The meta-analysis results supported the findings of the FinnGen study, showing that every 0.1 unit rise in ln-transformed retinol was associated with a 59% lower risk of IBD (OR: 0.41, 95% CI: 0.18 to 0.92, *P* =0.031) and a 72% lower risk of UC (OR: 0.28, 95% CI: 0.10 to 0.78, *P* =0.015). For absolute circulating β-carotene, we found no association with IBD, UC or CD in any database.

**Figure 3:**
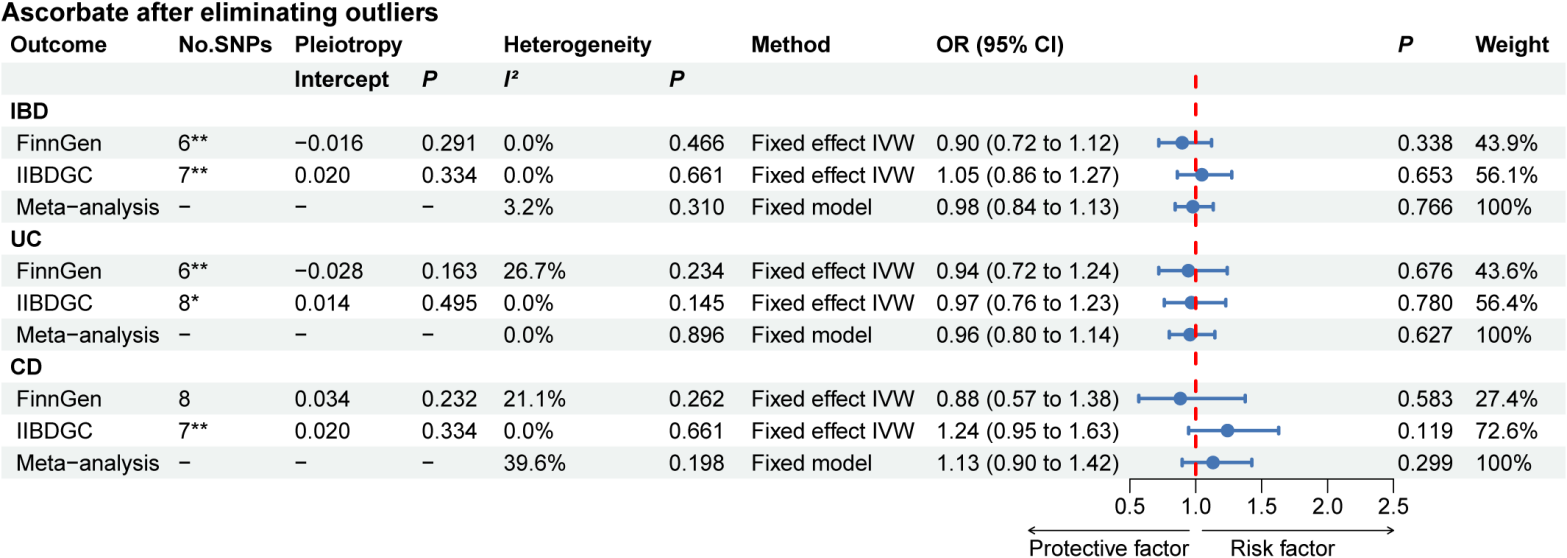
The MR analyses for absolute circulating ascorbate levels on the risk of IBD, UC, and CD after removing outliers. *: MR analysis results after removing one outlier by MR PRESSO; **: MR analysis results after removing two outliers by MR PRESSO. “-” represents not applicable. IBD, inflammatory bowel disease; UC, ulcerative colitis; CD, Crohn’s disease.

The results of the complementary MR analysis methods were in excellent agreement with the IVW results, indicating no causal link between the absolute circulating level of ascorbate and the risk of IBD, UC, and CD (**Supplementary Table 5**). For UC, two outlier SNPs were identified in FinnGen and one outlier SNP was identified in IIBDGC. For CD, while no outlier SNPs were detected in the FinnGen, two were identified in the IIBDGC. After eliminating outliers, there was also no indication of a causal association between the absolute circulating level of ascorbate and the risk of IBD, UC, and CD (**Supplementary Table 5**). Following the exclusion of the outliers, we re-performed meta-analysis and again found no evidence of causal association (**Figure 3**). To further confirm the causal relationship between the absolute circulating level of retinol in IBD and UC, we obtained relevant GWAS summary data from UK Biobank for replication analysis. The protective effect of retinol for IBD was not validated by replication analysis, but retinol still showed a protective effect for UC (**Supplementary Table 6**).

### 3.2 Circulating antioxidant metabolites and inflammatory bowel disease

**Figure 4:**
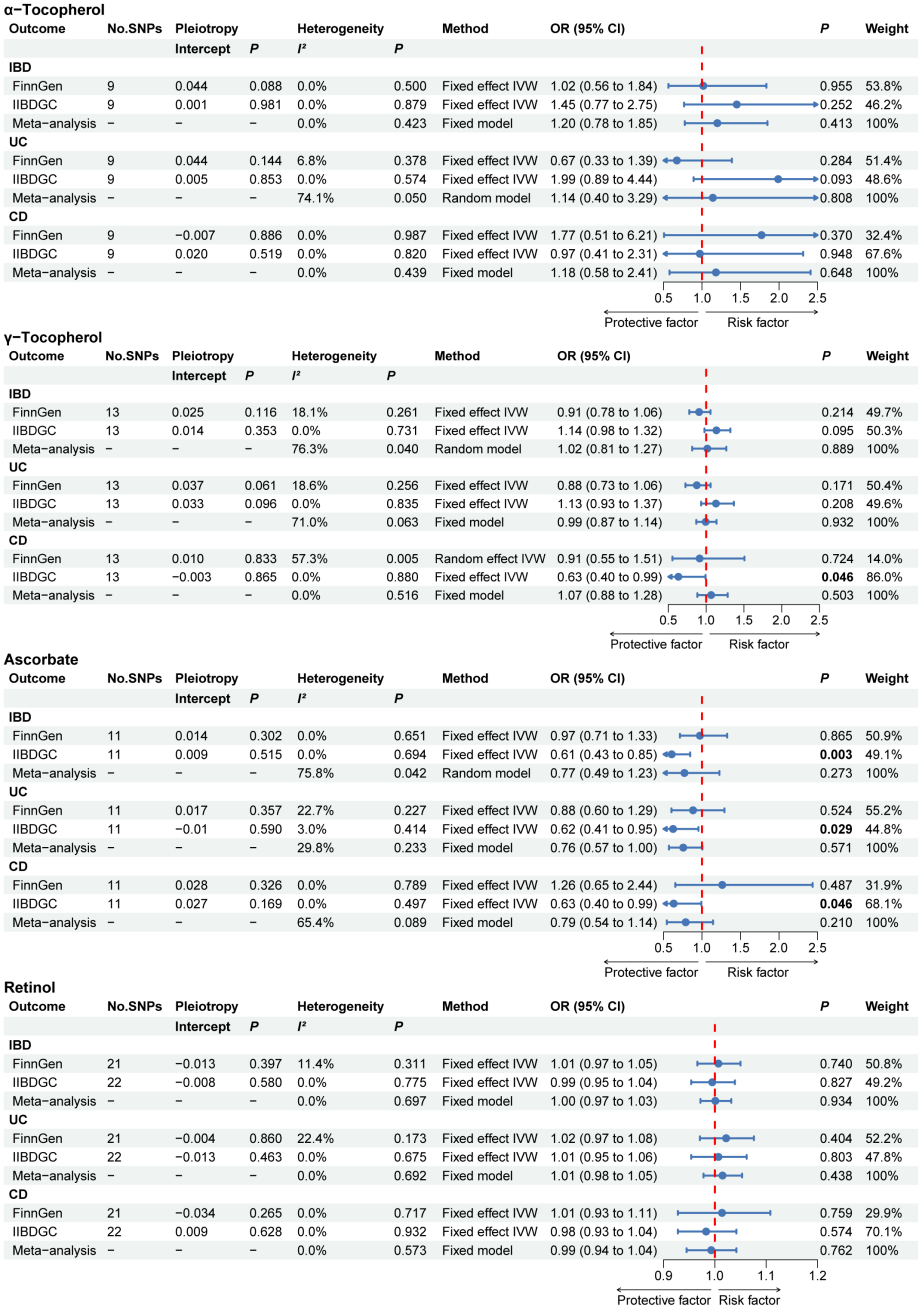
The primary MR analyses results of the causal effects of circulating antioxidant metabolites on IBD, UC, and CD. Significant and suggestive results highlighted in bold. “-” represents not applicable. IBD, inflammatory bowel disease; UC, ulcerative colitis; CD, Crohn’s disease.

The primary MR results for circulating antioxidant metabolites are shown in the forest plot in **Figure 4**. The range of the *P* value for the MR-Egger intercept test was between 0.061 and 0.981, suggesting the absence of horizontal pleiotropy. Although some evidence was found in single database, the meta-analysis results indicated no causal link between any of the circulating antioxidant metabolites and the risk of IBD, UC, and CD.

Similarly, the results of the complementary MR analysis methods were in excellent agreement with the IVW results, which did not show a cause association between circulating antioxidant metabolites and IBD, UC and CD (**Supplementary Table 7**). No outliers were found using the MR PRESSO method.

## 4 Discussion

In light of the rapid rise in IBD incidence in recent decades, identifying modifiable risk factors, such as diet, represents a promising avenue for preventing their progression. Instrumental variables for exposure were genetic variation from four absolute circulating antioxidant levels and four metabolite concentrations. The GWAS data for outcome was obtained from three large databases (IIBDGC, FinnGen, and UK Biobank). Using two-sample MR analysis, we found two causal associations, including absolute circulating retinol with IBD (*P* for meta-analysis: 0.031) and absolute circulating retinol with UC (*P* for meta-analysis: 0.015). Even though some other evidence was found in one database, it was considered incidental, since the overall analysis did not reveal a causal relationship between these antioxidants and outcome.

The association between antioxidants and the risk of IBD has been evaluated in a number of observational studies. However, it is controversial whether antioxidants can reduce risk of IBD. Retinol, a highly active form of vitamin A, serves as a key biochemical marker for vitamin A level in the human body. Using a double-blind method, Masnadi Shirazi and colleagues enrolled 150 patients diagnosed with UC with Mayo scores between 6 and 12 (26). The participants were assigned randomly to two groups: one receiving a daily dose of 25,000 IU of vitamin A and the other receiving a placebo. The intervention group exhibited a significant reduction in Mayo scores after the two-month supplementation period. Animal study have shown that supplementation with 5,000 IU of vitamin A significantly improved gut flora diversity and increased mucin expression in UC mice (27). The positive impact of vitamin A on disease activity in UC might be attributed to its influence on the equilibrium between Treg and Th17 cells (28). Retinoic acid increases the signaling of TGF-β and decreases inflammatory interleukin receptor levels, which could reduce the proliferation of Th17 cells (26). The main cause of inflammation in IBD is the release of the powerful proinflammatory cytokine IL-17 by Th17 cells (29, 30). A significant correlation exists between the serum levels of this cytokine and the disease activity in UC (31). Toll-like receptors on intestinal cells are activated by dietary antigens (32). This activation encourages dendritic cells (DCs) to exit the gut and travel to the mesenteric lymph nodes. Once there, they initiate the activation of T and B cells. When retinol is present, DCs stimulate the formation of regulatory T cells (33). Conversely, in the absence of retinol, DCs lead to the induction of Th17 cells, resulting in an inflammatory response characterized by IL-17 production. Moreover, impaired migration of B and T cells to the gastrointestinal tract was found in retinol-deficient rats (34). Retinoic acid enhances the generation of Treg cells, which play a role in counteracting colitis. The cytokine IL-10, secreted by these Treg cells, has the capacity to reduce inflammation (35). A study conducted in Japan, which included 384 patients with UC and 665 control subjects, revealed a negative correlation between retinol and the risk of developing UC (36). These results are similar to our findings. Kondo et al. (37) found that intraperitoneal injection of high-dose vitamin C (ascorbate) reduced the inflammatory response in DSS-induced UC model mice. Another study conducted at Mount Sinai Hospital, which included 20 patients with IBD, demonstrated that those with a deficiency in Vitamin C experienced more severe clinical symptoms (38). However, a retrospective study of 301 participants with IBD found no difference in clinical symptoms between those with and without vitamin C deficiency (14). According to a prior investigation carried out in France, the observation of vitamin C deficiency in IBD was probably due to lower sunlight exposure in different regions rather than as a result of the disease itself (39). The findings of a cross-sectional analysis involving 56 individuals diagnosed with UC revealed that increased consumption of lycopene was linked to reduced disease activity levels (40). Researchers also investigated the relationship between the levels of vitamin E (α-tocopherol, γ-tocopherol) and β-carotene and the risk of IBD (36, 41). The inconsistent results may be due to the fact that these reports are mostly from case-control or retrospective studies. In addition, observational research may be affected by unavoidable confounding factors that can lead to biased results. Specifically, these factors may act as biases that mask or exaggerate the relationship, thereby distorting some or all of the true relationship. Incorporating genetic variants into the MR design largely avoids the interference of confounding factors, thus providing relatively accurate estimates.

Oxidative stress is the result of a redox disequilibrium (42). The causal relationships identified by our MR analysis do not contradict with the assumption that oxidative stress plays a crucial role in the development of IBD. The circulating levels of some antioxidants, such as α-tocopherol, are not fully understood in terms of their antioxidant capacity. Nevertheless, we identified two associations that diet-derived antioxidants reduce the risk of IBD and UC. The strongest evidence is that the level of the absolute retinol is negatively associated with UC risk. The causal relationship between retinol and UC was significant not only in the combined database, but also in the UK Biobank. A suggestive association between retinol concentration and IBD was also found in the combined database, but did not pass validation in the UK Biobank.

The present study has three strengths. First, the two samples MR design reduces unnecessary risks that participants may face in clinical trials. Furthermore, the utilization of two distinct sets of instrumental variables for circulating antioxidants enhanced the informativeness of the MR findings. Third, we performed separate MR analysis in two large GWAS databases, and these results were pooled using meta-analysis to assess the overall effect. We also performed a replication analysis at UK Biobank.

Our study also has limitations. First, we obtained a limited number of SNPs for antioxidant instrumental variables from published GWAS data. However, these SNPs are situated in crucial genes related to antioxidant metabolism and are not associated with any of the other IBD risk factors in the PhenoScanner database (25). In the future, there is a need to identify more relevant loci through larger GWAS to increase the power of the instrumental variables. Second, there were no strictly defined IBD summary data available in UK Biobank, so we used self-reported IBD data for replication analysis, which may weaken the rigor of the results. Third, antioxidant concentrations are subject to considerable variation depending on the method of detection. There is a need to standardize detection techniques in the future.

## 5 Conclusion

Our study suggests that a higher absolute concentration of retinol is causally associated with a lower risk of UC. Present research raises uncertainties about whether higher levels of ascorbate, lycopene, or β-carotene in the blood are advantageous or harmful in the context of IBD. The circulating antioxidant metabolites are not causally related to the risk of IBD in our study. Therefore, in healthy adults without nutritional deficiencies, the use of these dietary antioxidant supplements to improve the concentrations of circulating antioxidant to prevent IBD is of limited clinical benefit. Future MR analyses are needed to extend the current findings based on large-scale GWAS using more SNPs as proxies for circulating antioxidants, especially retinol.

## Data availability statement

The original contributions presented in the study are included in the article/**Supplementary Material**. Further inquiries can be directed to the corresponding authors.

## Author contributions

MZ: Conceptualization, Writing – original draft. QL: Conceptualization, Data curation, Formal analysis, Writing – review & editing. WZ: Conceptualization, Data curation, Formal analysis, Writing – review & editing. JL: Conceptualization, Data curation, Formal analysis, Writing – review & editing. YZ: Funding acquisition, Writing – review & editing. YX: Funding acquisition, Writing – review & editing.
