# Supplementary material for: Diet-derived circulating antioxidants and risk of inflammatory bowel disease: a Mendelian randomization study and meta-analysis
Source: Front Immunol. 2024 Feb 21;15:1334395. doi: 10.3389/fimmu.2024.1334395 (PMC10915022; doi:10.3389/fimmu.2024.1334395)
Supplement: Supplementary file 1 [file DataSheet_1.pdf]

Supplementary Material

Diet-derived circulating antioxidants and risk of inflammatory bowel disease: A Mendelian randomization study

Menglong Zou[1], Qiaoli Liang[2], Wei Zhang[1], Junyao Liang[1], Ying Zhu[1*], and Yin Xu[1*]

*** Correspondence:**

Ying Zhu (zhuying_hunan089@163.com); Yin Xu (xuyin123@hnucm.edu.cn)

Supplementary Table 1 The summary information of the studies used for genetic instrumental variables extraction of circulating antioxidants.

Trait	Sample size	Age (years)	Sex (male, %)	Measurement method	Concentration
**Absolute circulating antioxidants**					
**Ascorbate (umol/L)**					
Fenland GWAS array	1,349	45±7.0	44%	Fluorometric assay	66.2±21.3
Fenland UKBB array	8,391	49±7.0	47%	Fluorometric assay	68.6±21.5
InterAct subcohort GWAS	3,521	51±9.0	35%	High-performance liquid chromatography with ultraviolet detection	42.8±19.0
InterAct subcohort core-exome	6,504	53±9.0	38%	High-performance liquid chromatography with ultraviolet detection	42.9±19.1
InterAct non-subcohort GWAS	2,944	55±8.0	48%	High-performance liquid chromatography with ultraviolet detection	36.4±17.6
InterAct non-subcohort core-exome	3,872	56±7.0	52%	High-performance liquid chromatography with ultraviolet detection	36.5±18.9
EPIC-Norfolk GWAS	16,756	59±9.0	47%	Fluorometric assay	53.8±20.2
EPIC-CVD subcohort	885	53±12.0	41%	High-performance liquid chromatography with ultraviolet detection	41.0±21.0

EPIC-CVD non-subcohort	6,765	57±8.0	55%	High-performance liquid chromatography with ultraviolet detection	37.9±20.8
**Lycopene (µg/dL)**					
HAPI	441	43.1±13.0	58%	Reverse-phase high-pressure liquid chromatography	39.2±19.9
**Retinol (µg/dL)**					
ATBC	4,014	58.1±5.0	100%	Reversed-phase liquid chromatography with diode-array UV detection	572 (796-654)
PLCO	992	64.6 ± 4.9	100%	Reversed-phase liquid chromatography with diode-array UV detection	672 (562-794)
**β-Carotene (µg/L)**					
NHS	2,344	58.8±6.4	0%	Reverse-phase high-pressure liquid chromatography	303±258
**Circulating antioxidant metabolites**					
**α-tocopherol**					
TwinsUK	5,966	53.4±14.0	7%	Liquid-phase chromatography and gas chromatography separation coupled with tandem mass spectrometry	NA
KORA	1,759	60.8±8.8	49%	Liquid-phase chromatography and gas chromatography separation coupled with tandem mass spectrometry	NA
**γ-tocopherol**					

TwinsUK	5,249	53.4±14.0	7%	Liquid-phase chromatography and gas chromatography separation coupled with tandem mass spectrometry	NA
KORA	977	60.8±8.8	49%	Liquid-phase chromatography and gas chromatography separation coupled with tandem mass spectrometry	NA
**Ascorbate**					
TwinsUK	518	53.4±14.0	7%	Liquid-phase chromatography and gas chromatography separation coupled with tandem mass spectrometry	NA
KORA	1,567	60.8±8.8	49%	Liquid-phase chromatography and gas chromatography separation coupled with tandem mass spectrometry	NA
**Retinol**					
TwinsUK	1,960	58 (32–87)	NA	The non-targeted metabolomics analysis was performed at Metabolon (Durham, North Carolina, USA) on a platform consisting of four independent ultra-high-performance liquid chromatography–tandem mass spectrometry instruments	NA

ATBC: Alpha-Tocopherol, Beta-Carotene Cancer Prevention Study; EPIC: European Prospective Investigation into Cancer and Nutrition; InCH: InCHIANTI Study; KORA: The Cooperative Health Research in the Region of Augsburg; NHS: Nurses' Health Study; PLCO: Prostate, Lung, Colorectal, and Ovarian (PLCO) Cancer Screening Trial; HAPI: Heredity and Phenotype Intervention Heart Study; NA, not applicable.

Supplementary Table 2 Instrumental variables associated with absolute circulating antioxidants.

Antioxidant	SNP	Effect allele	Other allele	EAF	*F*-statistic†	Beta	SE	*P*
Ascorbate	rs6693447	T	G	0.55	42.25	0.039	0.006	6.25E-10
	rs13028225	T	C	0.86	128.44	0.102	0.009	2.38E-30
	rs33972313	C	T	0.97	400.00	0.360	0.018	4.61E-90
	rs10051765	C	T	0.34	31.04	0.039	0.007	3.64E-09
	rs174547	C	T	0.33	26.45	0.036	0.007	3.84E-08
	rs117885456	A	G	0.09	42.25	0.078	0.012	1.70E-11
	rs2559850	A	G	0.60	93.44	0.058	0.006	6.30E-20
	rs10136000	A	G	0.28	32.65	0.040	0.007	1.33E-08
	rs56738967	C	G	0.32	34.31	0.041	0.007	7.62E-10
	rs9895661	T	C	0.82	62.02	0.063	0.008	1.05E-14
Retinol	rs10882272	C	T	0.35	56.25	-0.030	0.004	6.51E-15
	rs1667255	C	A	0.31	56.25	0.030	0.004	6.35E-14
Lycopene	rs7680948	A	T	0.20	40.11	-0.190	0.030	4.97E-09
	rs4635297	A	C	0.08	27.04	0.260	0.050	6.46E-07
	rs341075	A	G	0.02	26.19	-0.870	0.170	5.75E-07
	rs6108801	C	T	0.04	28.44	-0.480	0.090	4.07E-07
	rs2232315	A	G	0.03	24.34	0.740	0.150	1.26E-06
β-Carotene	rs6564851	G	T	0.36	98.67	0.149	0.015	1.60E-24

†The F-statistic for each SNP was calculated by the following formula: *F*-statistic = $Beta^2/SE^2$. EAF: effect allele frequency.

Supplementary Table 3 Instrumental variables associated with circulating antioxidant metabolites.

Antioxidant	SNP	Effect allele	Other allele	EAF	*F*-statistic†	Beta	SE	*P*
α-Tocopherol	rs10935814	A	G	0.10	19.87	-0.037	0.008	9.44E-06
	rs1404410	G	C	0.21	20.60	0.024	0.005	4.57E-06
	rs10245705	T	C	0.02	27.25	-0.066	0.013	1.95E-07
	rs11992435	G	A	0.05	20.63	-0.033	0.007	6.38E-06
	rs11145330	C	A	0.11	22.70	-0.032	0.007	1.95E-06
	rs7930821	T	C	0.02	19.98	0.067	0.015	7.53E-06
	rs261342	C	G	0.79	20.86	-0.017	0.004	5.41E-06
	rs1532701	A	G	0.55	20.85	0.014	0.003	5.07E-06
	rs10163969	T	G	0.04	19.36	-0.035	0.008	9.38E-06
	rs7238006	C	T	0.07	24.30	-0.028	0.006	6.77E-07
	rs2074731	A	C	0.17	22.26	-0.018	0.004	2.31E-06
γ-Tocopherol	rs6713914	C	T	0.43	26.13	-0.059	0.012	3.22E-07
	rs13069990	T	C	0.38	21.16	-0.051	0.011	4.44E-06
	rs6834631	G	T	0.04	23.93	-0.131	0.027	1.03E-06
	rs13103690	G	T	0.46	20.77	0.047	0.010	5.20E-06
	rs6826474	T	C	0.04	23.06	-0.138	0.029	1.56E-06
	rs2070006	C	T	0.63	20.90	-0.051	0.011	4.76E-06
	rs11167905	C	T	0.15	24.03	-0.080	0.016	9.83E-07
	rs9419004	C	G	0.19	20.35	-0.254	0.056	6.53E-06
	rs7112460	T	C	0.07	23.63	0.108	0.022	1.14E-06
	rs8057559	T	C	0.03	19.74	0.140	0.031	9.10E-06
	rs8105491	T	G	0.15	22.24	-0.070	0.015	2.30E-06
	rs808686	A	G	0.61	21.83	0.060	0.013	3.01E-06
	rs9606290	A	G	0.24	20.40	0.159	0.035	6.32E-06
	rs577596	A	G	0.33	24.74	-0.057	0.011	6.68E-07
Ascorbate	rs2794327	T	C	0.67	19.69	-0.036	0.008	8.78E-06
	rs6821770	A	G	0.14	19.52	0.038	0.009	8.92E-06

	rs10077932	T	C	0.14	21.35	-0.040	0.009	4.08E-06
	rs10520845	A	C	0.02	20.78	0.191	0.042	5.27E-06
	rs7038957	C	T	0.17	21.43	0.029	0.006	3.86E-06
	rs10492212	T	C	0.16	19.53	-0.027	0.006	8.66E-06
	rs10466757	T	A	0.84	19.66	-0.063	0.014	9.56E-06
	rs7350776	G	C	0.30	21.12	-0.024	0.005	3.86E-06
	rs261301	C	T	0.87	22.56	-0.032	0.007	2.06E-06
	rs13336771	A	C	0.17	20.15	0.062	0.014	7.39E-06
	rs1013104	T	C	0.44	21.16	-0.021	0.005	3.83E-06
	rs1060467	G	A	0.41	26.81	-0.023	0.005	2.61E-07
	rs5994305	G	A	0.17	24.52	-0.031	0.006	7.15E-07
Retinol	rs10019071	A	G	0.02	16.65	0.657	0.161	3.64E-06
	rs112293959	G	A	0.03	10.89	-0.429	0.130	5.70E-06
	rs114515641	G	T	0.03	10.30	0.414	0.129	7.12E-06
	rs1153379	A	G	0.93	15.14	-0.323	0.083	6.10E-06
	rs1176744	C	A	0.32	21.16	-0.207	0.045	3.50E-07
	rs118025446	A	G	0.03	17.49	-0.481	0.115	9.84E-06
	rs12955464	G	C	0.14	14.72	-0.234	0.061	3.71E-06
	rs139726207	G	A	0.04	11.31	0.370	0.110	4.46E-06
	rs149113848	G	C	0.01	13.23	-0.964	0.265	3.47E-06
	rs149478645	G	A	0.02	12.75	-0.507	0.142	1.30E-06
	rs17005512	C	G	0.17	13.20	-0.218	0.060	2.77E-06
	rs1842947	G	A	0.52	19.94	-0.192	0.043	8.34E-07
	rs2147337	G	T	0.66	12.89	0.158	0.044	9.01E-06
	rs2367816	G	A	0.77	19.99	0.228	0.051	9.46E-06
	rs2417325	T	C	0.93	15.34	0.329	0.084	1.29E-06
	rs3890033	C	T	0.38	11.76	0.144	0.042	8.56E-06
	rs3898702	T	C	0.20	16.15	-0.217	0.054	3.02E-06
	rs4135385	G	A	0.24	18.72	0.212	0.049	9.80E-06
	rs58411567	A	G	0.22	16.00	-0.208	0.052	3.02E-07
	rs6550239	A	G	0.74	14.54	-0.183	0.048	4.40E-06

rs75308833	T	C	0.02	11.29	-0.494	0.147	3.51E-06
rs7926028	T	G	0.45	10.33	-0.135	0.042	2.75E-06
rs945817	A	G	0.19	25.00	-0.275	0.055	6.46E-07
rs9586119	C	T	0.07	18.29	0.355	0.083	3.34E-06
rs117468033	T	C	0.01	26.13	-0.961	0.188	8.40E-06
rs568632536	T	C	0.03	14.39	0.531	0.140	8.08E-06

†The F-statistic for each SNP was calculated by the following formula: *F*-statistic = $Beta^2/SE^2$. EAF: effect allele frequency.

Supplementary Table 4 The summary statistics of the outcomes.

Trait	GWAS data source	Sample Size	Cases	Controls	Ancestry	Publication
IBD	IIBDGC	34,652	12,882	21,770	European	Liu, J.Z., et al. (1)
IBD	FinnGen	377,277	7,625	369,652	European	FinnGen study (2)
IBD†	UK Biobank	361,141	150	360,991	European	Pan-UKB project (3)
UC	IIBDGC	27,432	6,968	20,464	European	Liu, J.Z., et al. (1)
UC	FinnGen	376,564	5,034	371,530	European	FinnGen study (2)
UC	UK Biobank	361,194	2,143	359,051	European	Pan-UKB project (3)
CD	IIBDGC	20,883	5,956	14,927	European	Liu, J.Z., et al. (1)
CD	FinnGen	377,110	1,665	375,445	European	FinnGen study (2)
CD	UK Biobank	361,194	968	360,226	European	Pan-UKB project (3)

†Self-reported IBD, not a strict definition. IBD: inflammatory bowel disease; UC: ulcerative colitis; CD: Crohn's disease; IIBDGC: international inflammatory bowel disease genetics consortium.

FinnGen study: https://www.finngen.fi/en

Pan-UKB project: https://pan.ukbb.broadinstitute.org/

Supplementary Table 5 The complementary MR analyses results of the causal effects of absolute circulating antioxidants on IBD.

MR method	Ascorbate		
	No.SNPs*	OR (95% CI)	*P*
**FinnGen**			
**IBD**			
MR Egger	8	0.825 (0.360 to 1.892)	0.666
Weighted median	8	0.974 (0.756 to 1.256)	0.841
MR PRESSO†		0.956 (0.789 to 1.157)	0.644
**UC**			
MR Egger	8	0.941 (0.350 to 2.533)	0.909
Weighted median	8	1.056 (0.768 to 1.452)	0.736
MR PRESSO†		0.944 (0.674 to 1.321)	0.735
**CD**			
MR Egger	8	0.582 (0.268 to 1.264)	0.221
Weighted median	8	0.705 (0.384 to 1.296)	0.261
MR PRESSO†		NA	NA
**IIBDGC**			
**IBD**			
MR Egger	9	0.906 (0.525 to 1.564)	0.734
Weighted median	9	1.087 (0.859 to 1.375)	0.486
MR PRESSO†		1.046 (0.864 to 1.267)	0.644
**UC**			
MR Egger	9	0.775 (0.431 to 1.391)	0.421
Weighted median	9	0.970 (0.732 to 1.285)	0.832
MR PRESSO†		0.967 (0.710 to 1.316)	0.829
**CD**			
MR Egger	9	0.967 (0.419 to 2.234)	0.940
Weighted median	9	1.163 (0.843 to 1.603)	0.358
MR PRESSO†		1.241 (0.953 to 1.617)	0.109

Significant results highlighted in bold. †: No significant outliers. IBD: inflammatory bowel disease; UC: ulcerative colitis; CD: Crohn's disease; IIBDGC: international inflammatory bowel disease genetics consortium; NA, not applicable.

Supplementary Table 6 MR results for GWAS summary data from UK Biobank and antioxidant.

**Exposure**	**Outcome**	**Pleiotropy**		**Heterogeneity**		**MR method**	**No.SNPs**	**OR (95% CI)**	***P***
		**Intercept**	***P***	I^2	***P***				
Retinol	IBD	NA	NA	NA	NA	Wald ratio	1	1.000 (0.997 - 1.004)	0.848
Retinol	UC	NA	NA	NA	NA	Wald ratio	1	0.985 (0.973 - 0.997)	**0.016**

Significant results highlighted in bold. IBD: inflammatory bowel disease; UC: ulcerative colitis; NA, not applicable.

Supplementary Table 7 The complementary MR analyses results of the causal effects of circulating antioxidant metabolites on IBD.

MR methods	α-Tocopherol			γ-Tocopherol			Ascorbate			Retinol		
	No.SNPs	OR (95% CI)	*P*	No.SNPs	OR (95% CI)	*P*	No.SNPs	OR (95% CI)	*P*	No.SNPs	OR (95% CI)	*P*
**FinnGen**												
**IBD**												
MR Egger	9	0.249 (0.055 to 1.127)	0.114	13	0.668 (0.455 to 0.982)	0.065	11	0.711 (0.374 to 1.352)	0.325	21	1.055 (0.941 to 1.184)	0.370
Weighted median	9	1.036 (0.469 to 2.288)	0.930	13	0.798 (0.645 to 0.986)	0.036	11	0.855 (0.564 to 1.297)	0.461	21	1.034 (0.979 to 1.093)	0.233
MR PRESSO†		NA	NA		NA	NA		NA	NA		NA	NA
**UC**												
MR Egger	9	0.162 (0.025 to 1.025)	0.094	13	0.561 (0.355 to 0.889)	0.032	11	0.598 (0.244 to 1.470)	0.292	21	1.035 (0.888 to 1.207)	0.662
Weighted median	9	0.629 (0.239 to 1.653)	0.347	13	0.834 (0.636 to 1.094)	0.190	11	0.739 (0.423 to 1.288)	0.285	21	1.038 (0.967 to 1.114)	0.300
MR PRESSO†		NA	NA		NA	NA		NA	NA		NA	NA
**CD**												
MR Egger	9	2.217 (0.090-54.538)	0.641	13	0.805 (0.226 to 2.862)	0.743	11	0.673 (0.173 to 2.617)	0.581	21	1.147 (0.913 to 1.441)	0.253
Weighted median	9	1.609 (0.321 to 8.056)	0.563	13	1.025 (0.627 to 1.673)	0.923	11	0.869 (0.358 to 2.112)	0.757	21	1.033 (0.917 to 1.164)	0.593
MR PRESSO†		NA	NA		NA	NA		NA	NA		NA	NA
**IIBDGC**												
**IBD**												
MR Egger	9	1.427 (0.335 to 6.085)	0.645	13	0.969 (0.679 to 1.382)	0.863	11	0.496 (0.253 to 0.971)	0.071	22	1.025 (0.917 to 1.147)	0.667
Weighted median	9	1.932 (0.840 to 4.443)	0.121	13	1.150 (0.929 to 1.423)	0.200	11	0.559 (0.351 to 0.890)	0.014	22	0.997 (0.942 to 1.055)	0.914
MR PRESSO†		NA	NA		NA	NA		NA	NA		NA	NA
**UC**												
MR Egger	9	1.694 (0.273-10.524)	0.589	13	0.770 (0.489 to 1.214)	0.285	11	0.779 (0.319 to 1.903)	0.597	22	1.058 (0.919 to 1.219)	0.441
Weighted median	9	3.096 (1.003 to 9.559)	0.050	13	1.016 (0.773 to 1.334)	0.912	11	0.680 (0.384 to 1.203)	0.185	22	1.020 (0.950 to 1.095)	0.588
MR PRESSO†		NA	NA		NA	NA		NA	NA		NA	NA
**CD**												
MR Egger	9	0.527 (0.074 to 3.765)	0.544	13	1.135 (0.707 to 1.824)	0.610	11	0.347 (0.140 to 0.859)	0.048	22	0.949 (0.815 to 1.106)	0.511

Weighted median	9	0.879 (0.287 to 2.692)	0.821	13	1.190 (0.895 to 1.582)	0.231	11	0.548 (0.298 to 1.006)	0.052	22	0.966 (0.892 to 1.046)	0.390
MR PRESSO†		NA	NA		NA	NA		NA	NA		NA	NA

Significant results highlighted in bold.

†: No significant outliers. IBD: inflammatory bowel disease; UC: ulcerative colitis; CD: Crohn's disease; IIBDGC: international inflammatory bowel disease genetics consortium; NA, not applicable.

References

1. Liu JZ, van Sommeren S, Huang H, Ng SC, Alberts R, Takahashi A, et al. Association Analyses Identify 38 Susceptibility Loci for Inflammatory Bowel Disease and Highlight Shared Genetic Risk across Populations. *Nature genetics* (2015) 47(9):979-86. Epub 2015/07/21. doi: 10.1038/ng.3359.

2. Kurki MI, Karjalainen J, Palta P, Sipilä TP, Kristiansson K, Donner KM, et al. Finngen Provides Genetic Insights from a Well-Phenotyped Isolated Population. *Nature* (2023) 613(7944):508-18. Epub 2023/01/19. doi: 10.1038/s41586-022-05473-8.

3. Sudlow C, Gallacher J, Allen N, Beral V, Burton P, Danesh J, et al. Uk Biobank: An Open Access Resource for Identifying the Causes of a Wide Range of Complex Diseases of Middle and Old Age. *PLoS medicine* (2015) 12(3):e1001779. Epub 2015/04/01. doi: 10.1371/journal.pmed.1001779.
